# Randomized Trial of a “Dynamic Choice” Patient-Centered Care Intervention for Mobile Persons With HIV in East Africa

**DOI:** 10.1097/QAI.0000000000003311

**Published:** 2023-12-01

**Authors:** James Ayieko, Laura B. Balzer, Colette Inviolata, Elijah Kakande, Fred Opel, Erick M. Wafula, Jane Kabami, Asiphas Owaraganise, Florence Mwangwa, Hellen Nakato, Elizabeth A. Bukusi, Carol S. Camlin, Edwin D. Charlebois, Melanie C. Bacon, Maya L. Petersen, Moses R. Kamya, Diane V. Havlir, Gabriel Chamie

**Affiliations:** a Kenya Medical Research Institute, Kisumu, Kenya;; b University of California Berkeley, Berkeley, CA;; c Infectious Diseases Research Collaboration, Kampala, Uganda;; d University of California San Francisco, San Francisco, CA;; e National Institute of Allergy and Infectious Diseases, Bethesda, MD; and; f Makerere University College of Health Sciences, Kampala, Uganda.

**Keywords:** mobile, HIV retention, ART possession, viral suppression

## Abstract

**Background:**

Persons with HIV (PWH) with high mobility face obstacles to HIV care engagement and viral suppression. We sought to understand whether a patient-centered intervention for mobile PWH would improve viral suppression and retention in care, and if so, which subgroups would benefit most.

**Methods:**

In a randomized trial, we evaluated the effect of an intervention designed to address barriers to care among mobile (≥2 weeks out of community in previous year) PWH with viral nonsuppression or recent missed visits in Kenya and Uganda (NCT04810650). The intervention included dynamic choice of a “travel pack” (emergency antiretroviral therapy [ART] supply, discrete ART packaging, and travel checklist), multimonth and offsite refills, facilitated transfer to out-of-community clinics, and hotline access to a mobility coordinator. The primary outcome was viral suppression (<400 copies/mL) at 48 weeks. Secondary outcomes included retention in care and ART possession.

**Results:**

From April 2021 to July 2022, 201 participants were enrolled and randomized (102 intervention, 99 control): 109 (54%) were female participants and 101 (50%) from Kenya; median age was 37 years (interquartile range: 29–43). At 48 weeks, there was no significant difference in viral suppression in intervention (85%) vs. control (86%). The intervention improved retention in care (risk ratio: 1.06[1.02–1.1]; *P* < 0.001) and ART possession (risk ratio: 1.07[1.03–1.11]; *P* < 0.001), with larger effect sizes among persons with baseline nonsuppression and high mobility (≥2 weeks out of community in previous 3 months).

**Conclusions:**

Mobile PWH-centered care should be considered for high-risk mobile populations, including nonsuppressed and highly mobile PWH, to improve retention in care and sustain viral suppression over time.

**Trial registration:**

NCT04810650.

## Abstract 2

Supplemental Digital Content is Available in the Text.

## INTRODUCTION

As of 2021, 28.7 million of the 38.4 million (75%) persons with HIV (PWH) globally were on antiretroviral therapy (ART). Despite marked progress, 32% of PWH remain virally unsuppressed.^1^ Gaps to meaningful care engagement need to be identified and addressed to achieve the 95-95-95 UNAIDS goals to end the HIV epidemic.^2^ Mobility is a leading cause of disruption in HIV care engagement in sub-Saharan Africa and has been shown to negatively affect outcomes among PWH.^3,4^ Unpredictable travel schedules, as well as inflexible health systems that do not accommodate mobile patients, have been major contributing factors to poor health outcomes among highly mobile PWH.^5^ Mobility has been associated with increased risk of ART nonadherence, loss to follow-up from care, viral nonsuppression, development of drug resistance, and HIV-related death.^6^ There are sparse data on effective approaches to address the challenges posed by mobility in HIV care engagement. Interventions to address the barriers to care engagement that result from mobility are needed to improve HIV outcomes among mobile PWH.

To address this challenge, we sought to understand whether a patient-centered HIV care intervention for mobile PWH would improve viral suppression and retention, and if it did, which subgroups were most likely to benefit in rural Kenya and Uganda, within the SEARCH-SAPPHIRE trial (NCT04810650). Patient-centered care approaches have been shown to improve health outcomes by enhancing provider–patient interaction and enriching the care engagement experience by enabling care and treatment to be tailored to patient needs.^7,8^ In addition, we included participant choice in intervention components over time (ie, dynamic choice) as part of the SEARCH patient-centered care approach, allowing for heterogeneity in intervention delivery tailored to the needs of mobile populations.

## METHODS

### Study Design

During the pilot phase (Phase A) of the SEARCH-SAPPHIRE trial (NCT04810650), we conducted a two-arm randomized controlled trial to evaluate the impact of a patient-centered HIV care intervention to support retention in care, ART possession, and viral suppression among mobile PWH over a 48-week period. We also sought to determine which subgroups among mobile PWH benefited most from the intervention. In this setting, previous work by colleagues found frequent mobility (overnight travel) among PWH on ART, including work-related and non–work-related mobility in a cross-sectional survey. Furthermore, in this same survey, 9% of PWH on ART reported migration (change of residence) within the past 1 year, comprised interdistrict, intradistrict, and international migration.^9^ Our trial was 1 of 5 SEARCH-SAPPHIRE Phase A trials (NCT04810650): 3 evaluating dynamic choice HIV prevention delivery interventions^10^ and 1 evaluating a brief alcohol counseling intervention for people living with HIV with unhealthy alcohol use. The alcohol intervention trial was conducted in distinct communities and clinics from the present mobility intervention study.

### Recruitment, Randomization, and Masking

For inclusion in the study, we selected 5 rural, Ministry of Health–supported clinics in south-western Uganda and western Kenya. The study was conducted between April 15, 2021, and June 22, 2022. Participant enrollment into the study was based on the following inclusion criteria: (1) aged 15 years or older; (2) HIV-positive; (3) enrolled or new to HIV care in a study clinic regardless of duration on ART; (4) HIV RNA nonsuppression (>400 c/mL in the prior 12 months) as per their latest results or 2 missed visits in past 12 months; and (5) ≥2 weeks with nights spent away from home and outside the community during the 12 months before enrollment. We selected a 2-week period because patients typically visit clinic and receive ART refills every 1–3 months in this setting, and we considered ≥2 weeks away from home a period of mobility likely to disrupt ART access and adherence.

Consented participants were randomized to intervention or control by selecting a sequentially numbered scratch card, revealing the arm only when scratched by the participant. Participants were randomized 1:1 to the intervention and control arms using a stratified (country and sex) block design (random block sizes of 2 and 4) and followed up for a total of 48 weeks. Participants were not masked to the randomization group, but the study statistician (L.B.B.) was masked until trial completion.

### Intervention

For our study intervention, we assigned a mobility coordinator to each clinic who was responsible for intervention delivery. At baseline and during routine clinic visits, the mobility coordinator offered the intervention participants choice of the following options: (1) provision of a “travel pack,” which included an unmarked handbag (for women) or wallet (for men), along with an emergency 14-day supply of ART for abrupt unplanned travel (refilled at clinic visits on depletion), discrete alternative ART packaging options (eg, zip lock bags, envelopes, pill boxes) in an effort to address the stigma associated with carrying pill bottles (a marker of HIV positivity), and a travel checklist to remind participants of essential items to bring when traveling away from home for >1 night, including ART and the mobility coordinator's phone number; (2) inquiries about travel plans at each clinic visit; (3) “hotline” phone and SMS access to the mobility coordinator to discuss travel plans, with encouragement and a welcoming environment for communication of health needs during travel; (4) assistance in obtaining ART refills from out-of-community clinics for participants in need of refills during unexpected travel; (5) longer duration of pill dispensing, with up to 6-month supplies of ART; (6) out-of-facility refills for participants unable to return to clinic; and (7) assistance with transfers from the local clinic to out-of-community HIV clinics when participants out-migrated without a plan to return. Participants with unsuppressed viral load underwent intensive adherence counseling, and those who were suppressed were congratulated and encouraged to continue adhering to treatment. The mobility coordinator worked with clinic dispensaries to obtain antiretroviral medications for the emergency 14-day pill supply (in the travel pack) and longer ART refills for participants planning to travel. The mobility coordinator also worked with HIV service providers to assist with transfers of HIV care for participants who reported plans to out-migrate and requested transfer of care to another clinic.

### Control

Participants randomized to control received usual care and follow-up as delivered by the respective Ministries of Health, which included ART refills of up to 3 months duration, adherence counseling for persons with HIV viremia on last viral load measurement, and transfer to another clinic on patient request. No specific instruction or training was provided to clinicians.

### Primary and Secondary Outcomes

Our primary end point was viral suppression (HIV RNA <400 copies/mL) at 48 weeks of follow-up. Participants without viral suppression (HIV RNA ≥400 c/mL) or without an end point viral load measurement were treated as “failures,” while persons who withdrew consent, died, or formally transferred care to a nonstudy clinic were excluded from the primary analysis. The statistical analysis plan was full prespecified, including sensitivity analyses to assess the robustness of our analysis (see Table 1, Supplemental Digital Content, http://links.lww.com/QAI/C123).

Secondary outcomes for the trial were (1) time retained in care in days over 48 weeks, with nonretained time defined as the number of days starting >2 weeks from a missed clinic visit until return to clinical care and (2) ART-covered time over 48 weeks, defined as the proportion of trial follow-up days when a participant had a full regimen of ART. Retention was defined as proportion of time in care, with “out-of-care” time starting 14 days after a missed visit and ending with reengagement in care. Data for the primary and secondary outcomes (viral suppression, ART possession, and retention in care) were collected from Ministry of Health medical and pharmacy records, while data on the choice of intervention components were collected using study tools. To describe intervention implementation, we evaluated uptake, by measuring choice of intervention components offered at study visits at baseline, 12, 24, and 36 weeks after enrollment for intervention participants. We also evaluated intervention uptake at unscheduled encounters when participants reached out to a mobility coordinator outside of scheduled study visits and recorded sites of participant–mobility coordinator encounters for intervention delivery (ie, clinic-based, community-based, or mobile hotline-based encounters).

### Statistical Analysis

Outcomes were compared by arm with targeted minimum loss–based estimation, a method that adaptively selects the optimal adjustment variables to maximize precision, while maintaining nominal type-I error control under the null.^11,12^ In brief, we used 10-fold cross-validation to select from the following prespecified baseline variables: country, sex, age, care status (currently active in HIV care or not), mobility, baseline nonsuppression (HIV RNA ≥400c/mL), or nothing (unadjusted). The primary effect measure was the risk ratio (RR). We calculated two-sided 95% confidence intervals (CIs) and tested the null hypothesis that the intervention did not improve outcomes on the relative scale, with a one-sided test at the 5% significance level. Prespecified subgroups included country, sex, age group (15–30 years vs. 30+ years), HIV care status at enrollment, high mobility (defined as >14 nights outside the community in the 3 months before enrollment), alcohol use (any use in the 3 months before enrollment), and baseline viral nonsuppression (HIV RNA ≥400 c/mL). Based on a two-sample test of proportions, we estimated 200 participants (100/arm) would provide 80% power to detect a 20% or greater absolute difference in viral suppression from the intervention as compared with the control.

### Ethical Approval

We received ethical approval to conduct the study from the University of California, San Francisco Committee on Human Research, Makerere University School of Medicine Research and Ethics Committee, Uganda National Institute of Science and Technology, and the Scientific Ethical Review Unit of the Kenya Medical Research Institute (KEMRI). A written consent to participate in the study was provided by all participants before study enrollment.

## RESULTS

**FIGURE 1.**
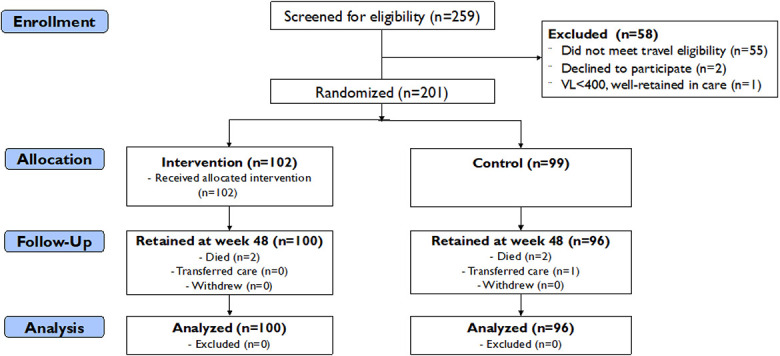
CONSORT diagram of a randomized controlled trial evaluating the SEARCH patient-centered care intervention vs. standard of care among mobile persons with HIV (PWH) with or at risk for HIV viral nonsuppression in Kenya and Uganda.

A total of 259 people were screened for eligibility, and of these, 58 were ineligible for trial participation (55 individuals did not meet travel eligibility criteria, 1 was well-retained in care, and 2 declined to participate; Fig. 1). Overall, 201 participants enrolled in the study, with 102 randomized to the intervention and 99 to standard care (control): 92 (46%) were male participants, and median age of the participants was 37 [Q1–Q3: 29–43] years (Table 1). Most participants were married; 132 (66%) and 142 (71%) had a primary school or lower level of education. Occupations reported by participants included farming (21%), fishing (17%), manual labor, and trade (13%). The median number of nights spent outside the community over the previous 12 months was 21 (interquartile range [IQR]: 14–60) days. Participants reported a median of 2 (IQR: 0–3) missed HIV care visits over the past 12 months because of travel. Overall, 91 (45%) participants had a viral load >400 copies/mL in the 12 months before enrollment (based on chart review), and 129 (64%) reported missing a dose of ART because of travel in the past 12 months. At baseline, 35 (17%) participants had viral nonsuppression (HIV RNA >400 c/mL).

**TABLE 1.** Characteristics of Participants Enrolled Into the Study, Overall and by Arm

	Intervention	Control	Total
	N = 102	N = 99	N = 201
Female, n (%)	56 (55)	53 (54)	109 (54)
Age, median [IQR]	38 [31, 42]	36 [29, 45]	37 [29, 43]
Country, n (%)			
Kenya	51 (50)	50 (51)	101 (50)
Uganda	51 (50)	49 (49)	100 (50)
Marital status, n (%)			
Single	10 (10)	8 (8)	18 (9)
Married	65 (64)	67 (68)	132 (66)
Widowed/divorced/separated	23 (23)	18 (18)	41 (20)
Cohabiting	4 (4)	6 (6)	10 (5)
Education, n (%)*			
Less than primary	9 (9)	2 (2)	11 (6)
Primary	69 (69)	62 (67)	131 (68)
Secondary	17 (17)	19 (20)	36 (19)
Postsecondary	5 (5)	10 (11)	15 (8)
Occupation, n (%)			
Farmer	23 (23)	19 (19)	42 (21)
Fishing	18 (18)	17 (17)	35 (17)
Manual labor/construction	16 (16)	11 (11)	27 (13)
Shopkeeper	8 (8)	9 (9)	17 (8)
Transport	8 (8)	4 (4)	12 (6)
Housewife	6 (6)	4 (4)	10 (5)
Other	23 (23)	35 (35)	58 (29)
Alcohol use, n (%)	37 (36)	36 (36)	73 (36)
Eligibility criteria, n (%)			
Unsuppressed VL in prior 12 mo	47 (46)	44 (44)	91 (45)
No VL in prior 12 mo	30 (29)	34 (34)	64 (32)
≥2 missed visits in prior 12 mo	77 (75)	71 (72)	148 (74)
New to HIV care	22 (22)	23 (23)	45 (22)
Mobility, median [IQR]			
Nights away prior 3 mo	4 [1,16]	4 [1,14]	4 [1,14]
Nights away prior 12 mo	22 [13,60]	21 [14,47]	21 [14,60]
Missed visits because of travel, median [IQR]†	2 [0,3]	2 [0,3]	2 [0,3]
Missed ART because of travel, n (%)	65 (64)	64 (65)	129 (64)
On ART, n (%)	98 (96)	95 (96)	193 (96)
INSTI-based regimen	78 (80)	77 (81)	155 (80)
NNRTI-based regimen	6 (6)	7 (7)	13 (7)
PI-based regimen	14 (14)	11 (12)	25 (13)
Baseline VL >400 c/mL, n (%)	20 (20)	15 (15)	35 (17)

* Missing among 8 participants (2 intervention and 6 control).

† Measured over prior 12 months and missing among 5 participants (3 intervention and 2 control).

Over 48 weeks of follow-up, 4/201 (2%) participants died (2 in intervention and 2 in control) and 1 participant in the control arm formally transferred care out of the community. Of the remaining 196 participants, comprising the primary analytic population, end point viral loads were obtained in 94% (185/196): 96% (96/100) in intervention and 93% (89/96) in control. Viral load was not measured in 11/196 (6%) participants (4 intervention and 7 control): 9 participants were found to have moved out of the study community during physical tracing, and 2 participants could not be found, and their whereabouts were unknown.

**FIGURE 2.**
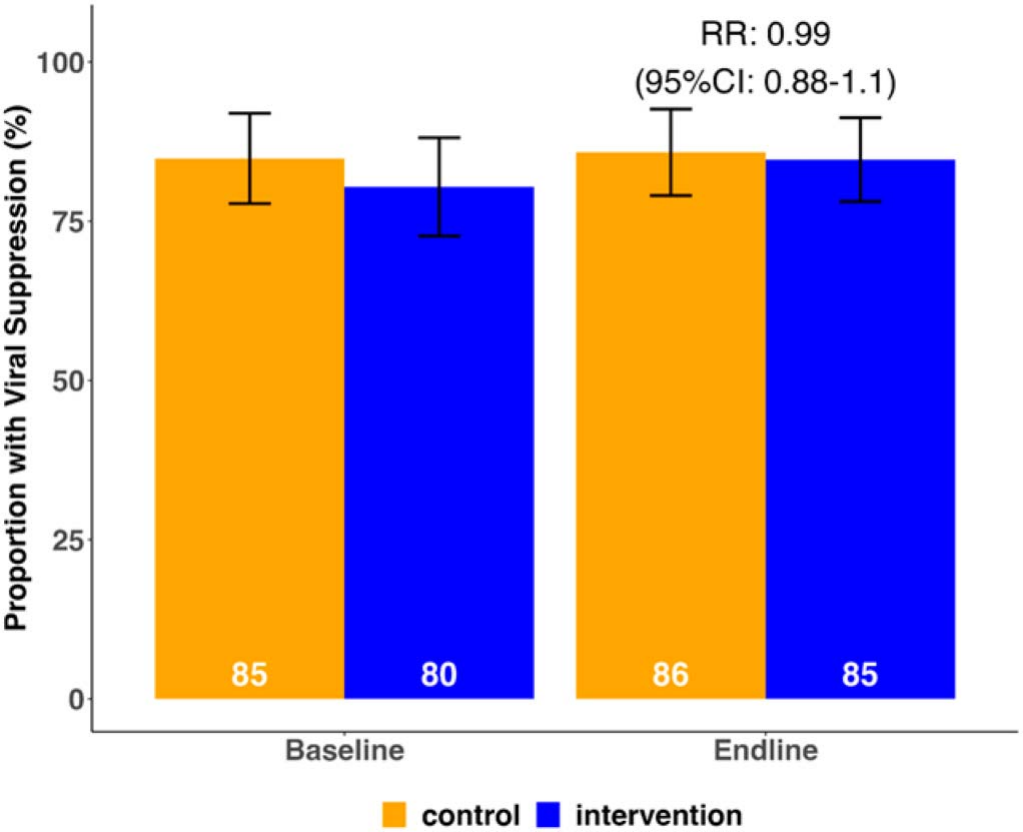
Proportion of participants with viral suppression (HIV RNA <400 copies/mL) by study arm at baseline and 48-week endline.

**FIGURE 3.**
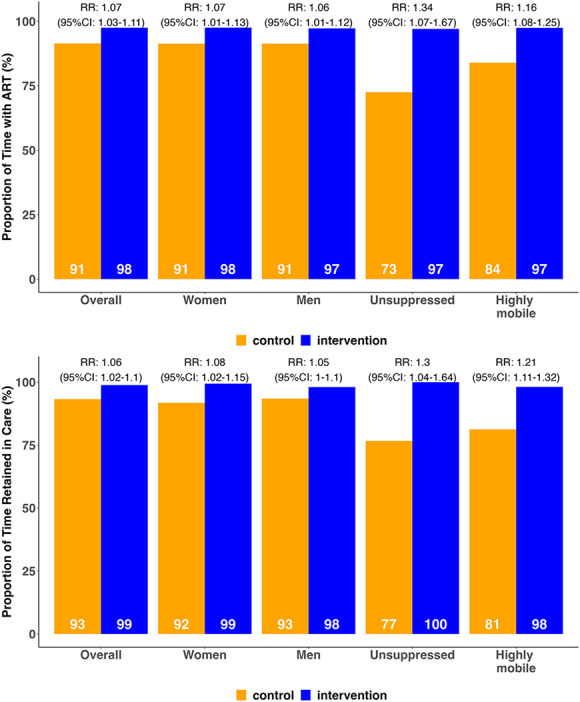
Proportion of time retained in care by arm (top) and time with ART by arm (bottom), overall, by country, and among participants with baseline unsuppressed viral load (>400 c/mL) and very high mobility (>14 nights outside of community in 3 months before enrollment).

HIV viral suppression at 48 weeks did not differ significantly by trial arm with 85% viral suppression in the intervention arm vs. 86% in the control arm, for a RR of 0.99 (95% CI: 0.88 to 1.10; *P* = 0.595, Fig. 2). No differences in viral suppression were observed across prespecified subgroups. All effect estimates were robust to prespecified sensitivity analyses, varying the handling of missing outcomes and covariate adjustment. Retention in HIV care over 48 weeks was significantly higher among intervention (99%) vs. control (93%) participants; RR: 1.06 (1.02–1.1), *P* < 0.001. Intervention effects on retention in care were observed across subgroups including country, sex, age group, alcohol use, baseline nonsuppression, and high mobility (Fig. 3). A larger effect size was observed among participants with baseline nonsuppression (retention in care of 100% intervention vs. 77% control; RR = 1.30 [95% CI: 1.04 to 1.64], *P* = 0.013) and high mobility (retention of 98% intervention vs. 81% control; RR = 1.21 [95% CI: 1.11 to 1.32], *P* < 0.001). ART possession was also significantly higher over 48 weeks in the intervention arm (98%) as compared with the control (91%); RR: 1.07 (95% CI: 1.03 to 1.11), *P* < 0.001. Intervention effects on ART possession were also observed across all subgroups, except for mobile PWH who were new to care (Fig. 3). A larger effect size was again observed among participants with baseline nonsuppression (ART possession: 97% intervention vs. 73% control; RR: 1.34 [95% CI: 1.07 to 1.67], *P* = 0.006) and high mobility (ART possession: 98% intervention vs. 84% control; RR: 1.16 [95% CI: 1.08 to 1.25], *P* < 0.001).

**FIGURE 4. A,:**
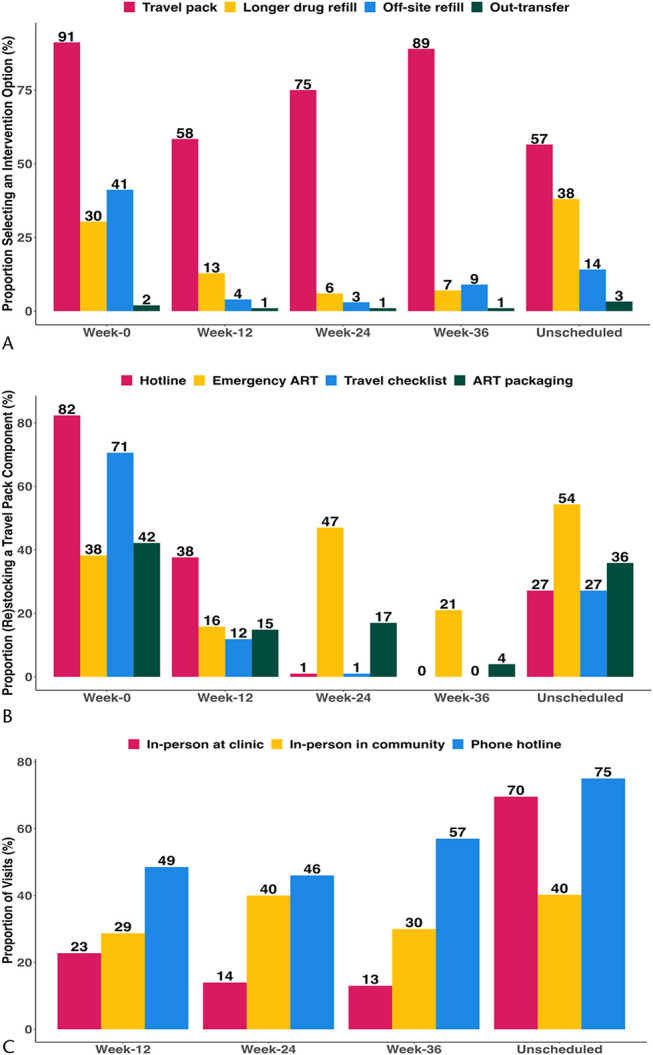
Uptake of patient-centered care intervention components as selected by participants over the 48-week study period, by study visit (ie, scheduled week postenrollment or unscheduled visit). B, Initial choice (week-0) and restocking choices for the “travel pack” intervention component of the mobile PWH-centered care intervention group participants, by study visit (ie, scheduled week postenrollment or unscheduled visit). C, Study visit site as selected by participants in the mobile PWH-centered care intervention group over the 48-week study period, by study visit (ie, scheduled week postenrollment or unscheduled visit).

Over the 48-week follow-up, all intervention participants selected at least 1 of the 7 mobility intervention options offered. Visit coverage was >98% over follow-up visits, and 90% of participants had at least 1 unscheduled visit. The choice of intervention options varied over time, with the travel pack being the most popular intervention component, followed by longer refills and out-of-clinic (“off-site”) refills (Fig. 4A). Assistance with transfer of care to clinics outside of the community was the least used intervention component. On initial (baseline) stocking and subsequent restocking of the “travel pack” component of the intervention, the emergency ART option remained popular throughout all visits, with changes in demand for the alternative packaging option seen over time (Fig. 4B). Approximately one-third of all scheduled study visits were conducted in person at the community (Fig. 4C). Phone hotline visits were the most popular both for scheduled (approximately half) and unscheduled visits (75%).

## DISCUSSION

A patient-centered HIV care intervention for mobile PWH did not increase viral suppression compared with standard care over 48 weeks but did increase retention in HIV care and ART possession among individuals with or at risk for HIV viral nonsuppression in Kenya and Uganda. Intervention effects on retention in care and ART possession were greatest among PWH who were unsuppressed at trial baseline and those with high mobility or alcohol use—key groups at increased risk of loss to follow-up and poor HIV treatment outcomes. These findings provide insights into which groups of mobile PWH are most likely to benefit from our patient-centered approach for mobile PWH and merit further evaluation. Exploring adherence challenges and addressing these through patient-centered treatment plans may be a next step in further improving HIV viral suppression rates among mobile PWH who are engaged in care but remain nonsuppressed.^7^

We did not observe a difference in viral suppression between participants who received the patient-centered intervention for mobile PWH versus standard care despite demonstrating a greater increase in viral suppression in the intervention compared with the control group from baseline to week 48 visit (5% increase from 80% to 85% vs. a 1% increase from 85% to 86%, respectively). We suspect that this finding may have been due to several factors. First, both groups started off at higher than anticipated HIV viral suppression rates (80%–85%), despite almost half (45%) having viral nonsuppression in the year before enrollment and three quarters having missed multiple HIV clinic appointments. High baseline viral suppression may have been a consequence of patients switching from non-nucleoside reverse transcriptase inhibitor (NNRTI)-based to dolutegravir (DTG)-based ART in the year or 2 before study start. Compared with NNRTIs, DTG is a more potent antiretroviral with a higher barrier to drug resistance. Multiple studies have shown that the transition from NNRTI to DTG-based ART has resulted in high levels of suppression among PWH retained in care, presumably in the context of varying adherence levels, and may explain the similar levels of viral suppression in both arms of our trial, despite significant differences in ART possession.^13–16^ Second, in response to the COVID-19 pandemic, clinics across the 2 regions moved toward multimonth dispensing of ART which diminished the difference between trial arms regarding the longer refill intervention component. Finally, it is possible that participants in the control group may have learned strategies from intervention participants in this individual randomized trial where participants could interact within clinics.

Our patient-centered HIV care intervention for mobile PWH increased retention in HIV care and ART possession compared with standard care, with the greatest intervention effects observed among subgroups who have previously been shown to be at increased risk of poor HIV outcomes,^17,18^ including PWH who were nonsuppressed and who were highly mobile. We also observed higher retention and higher ART possession among mobile PWH reporting alcohol use in the intervention arm compared with standard care. Previous studies have shown a strong association between alcohol use and poor retention in care and lower viral suppression relative to persons without alcohol use.^19,20^ Our findings demonstrate approaches that can improve engagement in HIV care in these high-risk groups, with potential long-term benefits not observed during a 48-week trial. Our findings suggest that the mobile patient-centered intervention should be considered for use among select subgroups of mobile PWH, including those with high mobility and HIV viral nonsuppression, rather than all mobile PWH.

Although viral suppression among participants was relatively high overall (85%) by the end of the trial, 15% of participants remained unsuppressed despite intensive adherence counseling in both arms, differentiated care (with multimonth dispensing in both trial arms), and high retention in care and ART possession in the intervention arm. We suspect that several factors contributed to this finding. First, ART adherence likely remained a challenge for participants who were unsuppressed at 48 weeks. Our findings suggest that there is an ongoing need for adherence interventions to supplement approaches for mobile PWH that improve HIV care engagement. Second, there may be multiple barriers hampering adherence such as intimate partner violence and food insecurity that highly mobile individuals face, in addition to the distinct challenges to care engagement that arise with high mobility.^21,22^ These may require even more flexible individualized needs assessments with dynamic, individualized responses to sufficiently address and support ART adherence and viral suppression.^7^

We observed high intervention uptake, with all intervention participants selecting at least 1 aspect of mobile PWH-centered care over 48 weeks. We offered choice of intervention options that could vary over time (ie, dynamic choice). Our findings demonstrate that need for intervention components was indeed variable, consistent with other studies that have identified care delivery that is responsive to patient preferences as a key element of patient-centered care.^23^ In our study, the travel pack was the most popular option used, possibly because of the multiple components it contained to address challenges such as forgetfulness, unplanned travel, or leaving home without one's pill bottles (addressed by providing a small stock of “emergency” ART) and discrete packaging to deal with stigma.

Our study has limitations. First, our duration of follow-up may have limited our ability to capture longer-term benefits expected with improved retention in care and ART possession over time, such as sustained viral suppression and avoidance of episodic viremia and the development of drug resistance. We also measured viral load twice—at baseline and trial endline—and could have missed periods of viremia that participants experienced in the interim period. Our participants had higher baseline viral load suppression than we anticipated which may have resulted in our inability to detect a difference in the primary outcome. We did not measure participant satisfaction or perceptions of the intervention in this analysis; however, qualitative interviews were conducted and will be shared in a future publication. Although we were able to measure travel pack refills (suggesting use of components such as the 14-day emergency supply of ART), we did not measure use of all intervention component options selected. Finally, we are unable to rule out contamination between the 2 arms because patients attending a clinic are likely to know and interact with each other and may have shared ideas about the intervention. To minimize this, we used mobility coordinators who only interacted with intervention group participants to deliver the intervention rather than HIV clinicians who interacted with both control and intervention participants.

In conclusion, our mobile PWH-centered intervention designed to overcome barriers to HIV care and viral suppression among mobile PWH significantly improved retention in care and ART possession, which may confer long-term benefit despite not showing a difference in viral suppression at 48 weeks. This intervention should be considered for specific high-risk mobile populations, such as persons with viral nonsuppression and high mobility, to improve retention in care and sustain viral suppression over time.

## Supplementary Material
