# Supplementary material for: Randomized Trial of a “Dynamic Choice” Patient-Centered Care Intervention for Mobile Persons With HIV in East Africa
Source: J Acquir Immune Defic Syndr. 2023 Dec 1;95(1):74–81. doi: 10.1097/QAI.0000000000003311 (PMC10695335; doi:10.1097/QAI.0000000000003311)
Supplement: Supplementary file 1 [file qai-95-74-s001.docx]

Sustainable East Africa Research in Community Health (SEARCH) Collaboration

Statistical Analysis Plan for

Dynamic Choice Care Intervention for Mobile Persons with HIV in Phase A of SEARCH-Sapphire

Laura B. Balzer, PhD1 and the SEARCH Study Team2

May 19, 2022 v1.0

1University of California, Berkeley

2[www.searchendaids.com](http://www.searchendaids.com/)

## Table of Contents:

1. [Study Overview 2](#_TOC_250006)
2. [Population and Characteristics 3](#_TOC_250005)
3. [Endpoint Measurement and Definition 3](#_TOC_250004)
4. [Evaluation of the SEARCH Intervention Effect 4](#_TOC_250003)
5. [Intervention Implementation 5](#_TOC_250002)

[Appendix: Power calculations 5](#_TOC_250001)

[References 6](#_TOC_250000)

# Study Overview

In Phase A of SEARCH-Sapphire (NCT04810650), we are conducting an individually randomized controlled trial to evaluate the effect of dynamic choice, patient-centered care intervention for mobile persons with HIV (PWH) in rural Kenya and Uganda. Details of the trial design and procedures can be found in the corresponding Study Protocol. Analyses plans for qualitative outcomes and cost-effectiveness outcomes are available elsewhere. Power calculations are given in the Appendix.

In brief, from April through July 2021, we enrolled 201 mobile PWH who were at risk of viral non-suppression. These participants were randomized to the intervention or the standard-of-care using a stratified random block design with stratification factors of country and sex and with random block sizes of 2 and 4. The randomization list was generated by an independent researcher.

The intervention includes choice of the following components to address barriers to HIV care among mobile PWH:

- - Travel pack, including emergency antiretroviral therapy (ART) supply, discrete

ART packaging, and a travel checklist

- - Hotline access to a mobility coordinator
  - Offsite and longer ART refills
  - Facilitated access to ART and HIV care outside of the community

The intervention is delivered by a mobility coordinator, stationed at each clinic, and follow-up is over 48 weeks.

## The primary objective is to evaluate if the intervention improved viral suppression (<400 copies/mL) among mobile PWH at week-48. Secondary endpoints, compared

between randomized arms, include retention in HIV care and ART possession. Additionally, within the intervention arm, we will report coverage and uptake of the intervention components at both scheduled and unscheduled visits.

# Population and Characteristics

The population of interest is PWH who are aged 15+ years, enrolled or new to HIV care, at risk of viral non-suppression (HIV RNA>400 copies/mL in the past year or 2+ missed HIV care visits in the past year), and mobile (2+ weeks out of the community in the past year).

To characterize measurement of this population, we will provide a participant flow diagram (i.e., a CONSORT diagram). Overall and stratified by trial arm and further by sex, we will summarize the baseline characteristics, including sex, age, country, marital status, occupation, education level, alcohol use (any use in past 3 months), mobility metrics (nights away in past 3 months and in past 12 months), impact of mobility on HIV care (missed visits and missed ART doses), baseline ART status and regimen, baseline viral suppression status, and trial enrollment criteria. We will categorize age as “younger” if aged 15-30 years and define persons to be “highly mobile” if they report spending >14 nights away from the community in the past 3 months.

# Endpoint Measurement and Definition

The **primary endpoint** is HIV viral suppression (HIV RNA<400 copies/mL) at 48-weeks. The primary analytic population consists of all living study participants who have not transferred HIV care to another health facility (as documented in clinical records). In other words, the primary analysis will exclude persons who died, withdrew, or transferred care, but will, otherwise, include persons regardless of their level of mobility or history of movement in/out of the community. In the primary analysis, missing endpoint viral loads will be treated as failures (i.e., unsuppressed). In pre-specified sensitivity analyses, described below, we will assess the robustness of these analytic choices.

Based on review of Ministry of Health medical and pharmacy records, we will also examine the following **secondary endpoints**:

- - Retention in care: the proportion of follow-up time where the participant is engaged in clinical care
  - ART possession: the proportion of follow-up time where the participant has a full regimen of ART

We will calculate total follow-up time for each participant as the number of days from their trial enrollment to their week-48 viral load measure. (For persons without a week- 48 viral load measure, we will use the close the endpoint ascertainment window.) Out- of-care time will start 14 days after a missed visit and end at re-engagement in care.

# Evaluation of the SEARCH Intervention Effect

We will assess the intervention effect with targeted minimum loss-based estimation (TMLE), which improves precision and power by adaptively adjusting for baseline outcome predictors.1–5 Here, we will use **TMLE with Adaptive Pre-specification** to flexibly control for baseline covariates, while maintaining Type-I error control and accounting for the randomization scheme.6–8 Using 10-fold cross-validation, we will chose the optimal approach for estimating the outcome regression (i.e., the expected outcome given the randomization arm and adjustment covariates) and the known propensity score (i.e., the conditional probability of being randomized to the intervention given the adjustment covariates). Specifically, we will select the combination of estimators (adjustment variables + approach) that minimizes the cross-validated variance estimate.

Our pre-specified, candidate adjustment variables consist of sex, age, country, being new to care at enrollment, baseline mobility (number of nights away from the community in the past 12 months), baseline viral suppression status, and nothing (i.e., unadjusted). Our pre-specified, candidate learners consist of generalized linear models (GLMs) adjusting for a single variable beyond the intervention indicator, stepwise regression, multivariate adaptive regression splines (MARS), MARS after screening based on outcome correlations, and the arm-specific mean outcome.

Primary effect estimates will be for the study sample and on the **ratio scale**:

1#𝑛∑"

�!(1) ÷ 1& ∑"

𝑌(0), where 𝑌(1) denotes the counterfactual outcome for

!#$

𝑛!#$ ! !

participant *i* under the intervention and �! (0) denotes the counterfactual outcome for

participant *i* under the control*.*9–11 Secondary comparisons will be on the difference

scale.

We will test the **null hypothesis** that the intervention did not improve viral suppression at week-48 using a one-sided test at the 5% significance level. We will also report point estimates and 95% confidence intervals for each effect measure and the arm-specific outcomes at baseline and week-48. Standard error estimation will be based on the estimated influence curve, and statistical inference will follow from the Central Limit Theorem (i.e., using the standard normal distribution).1

**Secondary analyses:** To assess the robustness of these findings, we will repeat these analyses using the unadjusted effect estimator. We will also repeat these analyses excluding persons with missing endpoints and using TMLE to adjust for missing endpoints.

**Subgroup analyses:** We will repeat these analyses within strata defined by country, sex, age group, mobility level, alcohol use, baseline viral suppression status, and trial enrollment criteria. In subgroups analyses, we will limit the candidate estimation approaches to main terms adjustment for a single covariate or the simple mean and use leave-one-out cross-validation for subgroups with <40 participants. To further understand effect heterogeneity, we may conduct variable importance measures (unadjusted and adjusted) to understand baseline predictors of endline viremia, overall and by arm.

**Secondary endpoints compared by arm:** We will implement analogous analyses to evaluate the intervention effect on retention in care and ART possession.

# Intervention Implementation

Within the intervention arm, we will describe coverage and uptake of the intervention at baseline (week-0), week-12, week-24, and week-36 study visits:

- - Visit coverage: number and proportion who attended study visits
  - Choice of intervention components: number and proportion* who selected the travel pack, longer ART refills, off-site refills, and/or facilitated out-transfer
  - Stocking and re-stocking of travel pack components: number and proportion* who selected the hotline, emergency ART, the travel checklist, and/or discrete ART packaging
  - Choice of visit type: number and proportion who had visits in-person at the clinic,

in-person in the community, or virtually on the mobile hotline

*Since participants could select more than one component, the proportion for a given visit-week may exceed 100%. Additionally, for unscheduled visits, we will report analogous metrics in terms of ever choice or use. We may also report summaries of the travel screen conducted by the mobility coordinator at each visit.

# Appendix: Power calculations

Sample size and power calculations were based on a two-sample test of proportions with *power.prop.test* function in *R*.12 We expect these calculations to be conservative,

because of the precision gained through stratified randomization, covariate adjustment during the analysis, and our pre-specified use of a one-sided hypothesis test.

We estimated 100 participants/arm would provide 80% power to detect at least a 20% absolute increase in viral suppression at 48-weeks from 40% under the standard-of- care. As shown in the following Figure, even with 25% attrition (from 100 to 75 participants/arm) and lower or higher than suppression in the control, these calculations suggest we would be well-powered to detect at least a 22.3% absolute increase in viral suppress.


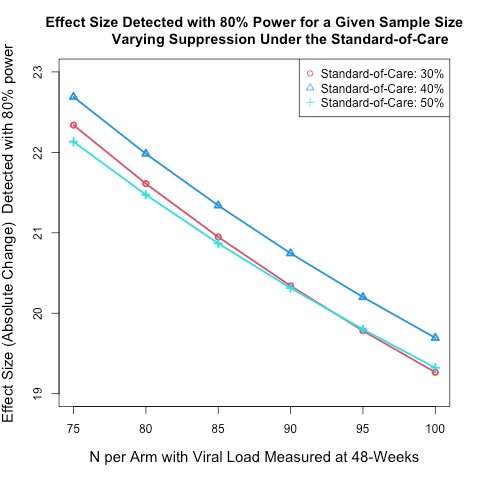


# References

1. van der Laan M, Rose S. *Targeted Learning: Causal Inference for Observational and Experimental Data*. Springer; 2011.
2. Moore KL, van der Laan MJ. Covariate Adjustment in Randomized Trials with Binary Outcomes: Targeted Maximum Likelihood Estimation. *Stat Med*. 2009;28(1):39-64. doi:10.1002/sim.3445
3. Rosenblum M, van der Laan MJ. Simple, Efficient Estimators of Treatment Effects in Randomized Trials Using Generalized Linear Models to Leverage Baseline Variables. *Int J Biostat*. 2010;6(1):Article 13. doi:10.2202/1557-4679.1138
4. van der Laan MJ, Rose S. *Targeted Learning in Data Science*. Springer; 2018.
5. Benkeser D, Díaz I, Luedtke A, Segal J, Scharfstein D, Rosenblum M. Improving precision and power in randomized trials for COVID-19 treatments using covariate adjustment, for binary, ordinal, and time-to-event outcomes. *Biometrics*. 2021;n/a(n/a):1-15. doi:10.1111/biom.13377
6. Balzer L, van der Laan MJ, Petersen M, SEARCH Collaboration. Adaptive Pre- specification in Randomized Trials With and Without Pair-Matching. *Stat Med*. 2016;35(10):4528-4545. doi:10.1002/sim.7023
7. Havlir DV, Balzer LB, Charlebois ED, et al. HIV Testing and Treatment with the Use of a Community Health Approach in Rural Africa. *N Engl J Med*. 2019;381(3):219- 229.
8. Balzer LB, van der Laan M, Ayieko J, et al. Two-Stage TMLE to Reduce Bias and Improve Efficiency in Cluster Randomized Trials. *Biostatistics*. 2021;kxab043. https://doi.org/10.1093/biostatistics/kxab043
9. Neyman J. Sur les applications de la theorie des probabilites aux experiences agricoles: Essai des principes (In Polish). English translation by D.M. Dabrowska and T.P. Speed (1990). *Stat Sci*. 1923;5:465-480.
10. Rubin DB. Comment: Neyman (1923) and Causal Inference in Experiments and Observational Studies. *Stat Sci*. 1990;5(4):472-480.
11. Balzer LB, Petersen ML, van der Laan MJ. Targeted estimation and inference of the sample average treatment effect in trials with and without pair-matching. *Stat Med*. 2016;35(21):3717-3732. doi:10.1002/sim.6965
12. R Core Team. *R: A Language and Environment for Statistical Computing*. R Foundation for Statistical Computing; 2022. [http://www.R-project.org](http://www.R-project.org/)
