# Supplementary material for: Randomized Trial of a “Dynamic Choice” Patient-Centered Care Intervention for Mobile Persons With HIV in East Africa
Source: J Acquir Immune Defic Syndr. 2023 Dec 1;95(1):74–81. doi: 10.1097/QAI.0000000000003311 (PMC10695335; doi:10.1097/QAI.0000000000003311)
Supplement: Supplementary file 2 [file qai-95-74-s002.docx]

**Supplementary Table 1.** Baseline covariates included in the adjustment set in the primary analysis for each endpoint. Selection was done in a pre-specified but adaptive way to maximize precision without inflating Type-I error control. See the Statistical Analysis Plan for details.

| **Analysis** | **Subgroup** | **Adjustment variables** |
| --- | --- | --- |
| Primary outcome: viral suppression (VS) | Overall | Country, baseline viral suppression (VS) |
| Secondary outcome: retention in care | Overall | Age |
|  | Women | Country, baseline mobility |
|  | Men | Baseline mobility, baseline VS |
|  | Unsuppressed | Baseline mobiliity |
|  | Highly mobile | Baseline VS, baseline care status |
| Secondary outcome: adherence to antiretroviral therapy | Overall | Baseline mobility, baseline VS |
|  | Women | Age, baseline mobility |
|  | Men | Baseline mobility, baseline VS |
|  | Unsuppressed | No adjustment |
|  | Highly mobile | Baseline mobility, baseline VS |
